# Topics of Public Interest

**Published:** 1904-04

**Authors:** 


### TOPICS OF PUBLIC INTEREST.

### Status of Osteopathy.

[From *Saint Louis Clinique*, Editorial, March, 1904.]

A GLANCE at the medical laws recently adopted, or introduced in the legislatures of several states, shows that there is a strong tendency on the part of legislatures to make changes in the medical laws looking to a better recognition of all the side issues of the practice of medicine. This is especially marked when we take up the subject of osteopathy. In many states this cult is dignified as a system of practice, and while it has certain limitations it still holds a position before the law equally as high as does the practice by the physician who has devoted a lifetime to the study of his profession, and who, in addition to the knowledge acquired by the osteopath, is master of the entire field of medical science. It is a disposition to make a part, and a very small part at that, equal to the whole.

This tendency on the part of legislatures is due to two principal causes: first, to the utter apathy of the medical profession and the want of organised effort on behalf of sound medical laws; second, to the aggressive character of the promoters of this special brand of sectarianism.

From the Report of the Illinois State Board of Health for 1903, we quote the following: "osteopathy (osteon—a bone, pathos, suffering), 'a system, method or science of treating diseases of the human body,' is legalised and its practice is regulated by legislative enactments in the following states—Arizona, Arkansas, California, Connecticut, Indiana, Iowa, Kansas, Michigan, Minnesota, Missouri, Montana, Nebraska, New Mexico, North Carolina, North Dakota, Ohio, Oklahoma, South Dakota, Tennessee, Vermont, Virginia, and Wisconsin. In Massachusetts and Texas osteopaths are exempt from the provisions of the medical law of the state." In Illinois bills have been passed by the two last legislatures legalising osteopathy in the state, but these have been vetoed by the governor in each case,—the first by Governor Tanner in 1897; and the last by Governor Yates in 1903.

Quoting again from the Report of the Illinois State Board of Health we see that "under the provisions of the medical law of Illinois enacted in 1899, the State Board of Health is empowered to examine and license persons who desire to practise any certain 'system or science of treating human ailments, who do not use medicine internally or externally and who do not practise operative surgery,' the said examination to 'be of a character sufficiently strict to test their qualifications as practitioners.'"

This would appear to be a fair law. It does not prohibit the practice of osteopathy or any other method of healing the sick. It only asks that persons who assume to do this shall show that they possess certain qualifications. It does not ask of the osteopath as much as it does of the one who makes use of all the therapeutics known to the science of medicine. In spite of this very liberal law we shall see a bill introduced in the next legislature which will seek to relieve the osteopath of even this examination, or to establish a separate board of examiners for them.

The whole matter will resolve itself into this one fact; a certain class of men are determined to get all the rights and privileges of the doctor without the necessity of earning those privileges by a thorough and systematic study of the science of medicine. A bill is being pushed in the Kentucky legislature providing for a special board of examiners for osteopaths, and the whole strength of that power will be used to secure its passage.

The medical profession should not be indifferent to this matter, not because the results will be particularly disastrous to it, but for the better reason that the medical profession is better qualified to judge what is best for the public. The medical profession has ever been the most earnest advocate of measures for the relief and prevention of disease, and should not now be willing to permit unqualified persons to become the guardians of this particular part of the public interests. The profession of medicine is too liberal to attempt to persecute any sect in medicine,—a charge frequently brought up when steps are taken looking to the betterment of the condition of those who are unable to judge for themselves in matters affecting health,—but it should take such steps as are necessary to secure legislation which will prevent unscrupulous persons from imposing upon the ignorant, and especially should it be seen that no person is permitted to practise medicine under the cloak of osteopathy or any other so-called science except according to fixed standards.

---

### Recent Progress in Matters Pertaining to the Care of the Insane in the State of New York.

SOME of the measures of improvement in the care of the insane of the State of New York carried through during the past few months by the Lunacy Commission and formulated by that commission are stated in the following terms:

1. The Pathological Institute has been reorganised and more than sixty of the medical men connected with the staffs of the fourteen state hospitals have been instructed recently at the institute on Ward's Island in the later development of psychiatry along clinical, pathological, psychological and clinical lines. The legislative appropriation for the institute is now $25,000 annually.

2. The hospitals have been opened to medical internes in the same manner as general hospitals. Year before last sixteen clinical assistants entered the service in this way, and last year the number was about thirty.

3. The legislature recently passed the lunacy commission's bill for the appointment of a medical inspector to insure a more thorough inspection of the thirty-nine institutions under its charge —namely, 23 private retreats, 2 criminal asylums, and 14 state hospitals for the insane. Such inspection, especially of the private asylums, in which there are about 1,000 patients, has never been adequate.

4. To remedy overcrowding the lunacy commission proposes to construct a new hospital in the territory north of Albany and Troy on the colony system. A scheme similar to that of the Craig Colony for epileptics will be carried out. The site will be selected and plans made this year. This colony for 1,500 to 2,000 patients should be ready inside of two or three years.

5. Three tuberculosis hospitals, each with a hundred beds were put under construction last summer at a cost of $90,000, located at Middletown, Utica and Binghamton on the grounds of the state hospitals at those points; and the plans made by Dr. Peterson and the state architect embody the main features of such hospitals described in the King Edward Prize Essays. In the meantime tent life for the tuberculous insane has been in vogue at the Manhattan State Hospital, East (under Dr. MacDonald), for two or three years, and for a shorter time at Binghamton and other of the state hospitals.

6. The country colony for a few of the working classes of the insane, as an offshoot of the Utica State Hospital, has been enlarged. A similar colony has been established at the Willard State Hospital and two are in existence at the state hospitals at Binghamton and Poughkeepsie.

7. A new departure last year was the creation of a summer camp for between 40 and 60 insane on the lake shore about 15 miles from the Rochester State Hospital, which was put in operation to the great delight of both patients and attendants.

8. The feature of nurses' homes having been found so useful at some of the hospitals two additional ones were put up last summer, one at Kings Park State Hospital, Long Island, for 300, and one at the Gowanda State Hospital for 100 nurses.

9. Six or seven residences for superintendents and separate houses for the medical staffs were put up last season at as many of the state hospitals, thus removing the officials from the central main buildings and utilising the vacated space for patients.

10. A bill providing for emergency commitments, recommended by the lunacy commission, was passed by the legislature a year ago. Copies of this law have been sent to all the examiners in lunacy of the state. It is believed that this will mean great good to the insane and prevent the all too frequent incarceration of urgent cases in jails and station houses.

11. The improved ration brought about during the past year, though entailing an additional cost to the state of a hundred thousand dollars a year or more, has added greatly to the comfort of the patients and to the satisfaction of the medical officials and various visiting boards.

12. A strong effort is making by the lunacy commission to increase the number of deportations of alien insane, and through the efforts of New York State the federal government passed a law making the limit three years instead of one; that is, an immigrant becoming insane within three years after landing in the United States may be returned to his own country.

13. The movement for the establishment of reception hospitals for acute curable cases in the large cities has gained strength. While the bill for the psychopathic hospital for New York city, which was to be the first of several such reception hospitals, failed to pass last year, it is expected that success will attend the commission's efforts during the present session.

For these and still other reasons the commission in lunacy is confident of much improvement in the care of the insane in the very near future. It is proper to add that many of the changes alluded to in the foregoing statement have become accomplished facts, since the issuance of this circular several months ago.

---

### The Antitoxin Treatment and its Manufacture.[1]

*Editor Buffalo Medical Journal:*

SIR—Upon the subject of the early antitoxin treatment of diphtheria your views doubtless coincide with those of the leading teachers of medicine in this country and Great Britain; and, since you are familiar with the latest literature, we venture to act on the repeated suggestion of our medical advisers and ask your assistance in disseminating certain truths which are unassailably sound and correct, but which have by no means the vogue that they deserve.

McCollom, of Boston, (*Boston Medical and Surgical Journal,* Dec. 25, 1900,) expressed the opinion three years ago that "small doses are of little avail in the treatment of grave types of the disease;" that "in order to obtain the best results the serum must be heroically administered." The same writer states that heart complications of a serious nature have not been so frequent among the thousands of patients treated in a Boston hospital, nor has paralysis been so prominent. Finally, "since the larger doses of antitoxin have been given, the death rate has been materially reduced; the reduction having occurred among the apparently moribund cases."

1. NOTE BY THE EDITOR.—So much of interest attaches to the antitoxin treatment at the present time, and again so much controversy is taking place just now, we prefer to let expert and reliable manufacturers speak for themselves in relation to these questions. The communication of Messrs. Parke, Davis & Co. is so clear and intelligent that we are pleased to present it under "Topics of Public Interest," rather than under "Correspondence."

The *British Medical Journal* some time since, (Nov. 10, 1900,) advised the injection of 2,000 UNITS IN MILD CASES, and 4,000 to 10,000 units in cases not mild,—that is "if either of the tonsils is entirely covered with thick membrane, or the palate, the nasal passages, or larynx is attacked, or if there be enlargement of glands, fetor, increased frequency of pulse, albuminuria, pyrexia and restlessness." In "bad" cases, 16,000 to 20,000 units in 24 hours will be required.

*Immunisation.*—J. Dutton Steele, M. D., of the University of Pennsylvania, (*Therapeutic Gazette,* July 15, 1901,) cites 17,516 immunised cases collected by Biggs, of which only 129 developed mild diphtheria in thirty days: twenty patients were attacked after thirty days; and in all THERE WERE BUT TWO DEATHS.

There seems to be a variety of opinion as to the duration of the artificial immunity thus induced, but at all events the tendency is unquestionably in the direction of large doses. As the author just quoted says: The dose (immunising) which was originally placed at 200 to 500 units has advanced so that now from 500 to 1,000 units are more frequently given." It is a well-known fact that the Klebs-Löffler bacillus may be and has been repeatedly found in the throats of immunised nurses and others exposed to diphtheria, without the appearance of clinical symptoms of the disease.

*Stamping out Epidemics.*—In order to check the spread of diphtheria in any densely populated community, two measures are essential—namely, isolation, and immunisation by the injection of 1,000 units of antidiphtheritic serum. Inasmuch as the micro-organisms have been found in the throats of convalescents for two weeks after recovery, there is always a possibility that their virulence may survive the period of immunity conferred by a small prophylactic dose of serum. Hence, the great importance of using a sufficient amount of this harmless agent whenever necessary to employ it as a preventive measure. There is every reason to believe that the initial immunising dose for a child under ten years should be at least 1,000 units, and when the patient's environment subjects him to repeated exposure the injections should be given at intervals of not less than three weeks.

*Municipalities as Manufacturers.*—There is another aspect of this subject that is worthy of the thoughtful consideration of the medical editor, teacher and practitioner. Recent agitation by the newspapers has prompted the health officers of some cities to venture to undertake the manufacture of antitoxin. That this is a dangerous procedure has been demonstrated, notably in the case of the city of Saint Louis. A safe antitoxin cannot be made successfully except under the most favorable conditions. The essential details of a delicate scientific process cannot be entrusted to stable boys or unskilled laborers. Only perfectly sound animals can be utilised. They must be housed in large, airy, well-lighted and drained buildings, equipped with aseptic fittings. They must be cared for with scrupulous fidelity to the principles of hygiene, and must be under the constant care of trained veterinarians. The appointments of the laboratories must be perfect. The appliances must be absolutely sterile during the entire process. This involves a vast outlay of capital and the employment of high-salaried and thoroughly trained men. Our own equipment, including our great twin stables, represents an outlay of nearly half a million dollars.

*Federal Supervision.*—The United States Government recognises the importance of a perfect equipment for this work, and now, after a thorough inspection by the Bureau of Public Health and Marine Hospital Service, issues a license to those manufacturers who can comply with its rigid requirements. It is needless to say that municipal laboratories, not being engaged in interstate commerce, do not come under the jurisdiction of United States laws, and therefore do not have the benefit of rigid government inspection,—an inspection so severe that, rather than undergo the expense necessary to comply with the requirements of the United States Government, a number of small concerns have withdrawn from the industry.

Apropos of the subject of the present method of classifying serum under one grade, you may be interested in the remarks of Dr. B. R. Shurly, of Detroit, which appeared in a recent issue of *Pediatrics.* The writer says:

A fatal error has frequently come under my observation, that of mistaking the meaning of the classification, double X serum. Double X serum refers to twice the bulk or concentration of the product, but no increase in antitoxic value. In other words, 2,000 units single X are identical in curative value with 2,000 units double X. The majority of physicians who have used antitoxin occasionally fall into the serious and fatal error of believing one to be twice the value of the other. It would seem necessary to request the laboratories manufacturing antitoxin to make the directions and title of the product so clear that no possible confusion can arise. Under the present system the mistake is leading to fatal results.

We submit these facts because they are of more than ordinary interest just now. We trust you will find them worthy of serving as the theme of a timely editorial in an early issue of your periodical.

PARKE, DAVIS & CO.

Detroit, February, 1904.

